# Analysis of the Upregulated Expression Mechanism of Apoptotic Chromatin Condensation Inducer 1 in Hepatocellular Carcinoma Based on Bioinformatics

**DOI:** 10.5152/tjg.2024.23454

**Published:** 2024-04-01

**Authors:** Yulian Tang, Anni Ni, Lishuang Sun, Shu Li, Genliang Li

**Affiliations:** 1 Youjiang Medical University for Nationalities School of Laboratory Medicine, Baise, Guangxi, China; 2 Youjiang Medical University for Nationalities Graduate School, Baise, Guangxi, China; 3 Youjiang Medical University for Nationalities School of Basic Medical Sciences, Baise, Guangxi, China

**Keywords:** Acin1/Acinus, gene expression, ceRNA, hepatocellular carcinoma

## Abstract

**Background/Aims:**

A large number of differentially expressed molecules exist in hepatocellular carcinoma (HCC), and the mechanism by which they upregulate or downregulate expression is still unclear. The purpose of this study is to explore the possible mechanism of differential expression of apoptotic chromatin condensation inducer 1 (Acin1) in HCC.

**Materials and Methods:**

A mouse HCC model was constructed, and the expression of *Acin1* in HCC was analyzed by whole transcriptome sequencing, bioinformatics analysis, and reverse transcription-quantitative polymerase chain reaction, and differentially expressed *Acin1*-related genes were screened to construct a protein–protein interaction and competing endogenous RNA (ceRNA) network. The microRNA (miRNAs) targeting *Acin1* were further predicted using online databases and finally compared with sequencing data.

**Results:**

The expression of *Acin1* was significantly up-regulated in HCC compared to the paracancerous and healthy control groups (*P* < .001). The top 10 upregulated genes closely related to *Acin1* (*Slc3a2*, *Wiz*, *Srrm2*, *Akt1*, *Hnrnpu*,* Sap18b*, *Pabpn1*,* Ddx39b*, *Eif4a3*, and *Rnps1*) were mainly involved in pathways such as messenger RNA (mRNA) surveillance, RNA transport, spliceosome, Janus kinase/signal transducers and activators of transcription signaling, apoptosis, and ubiquitin-mediated proteolysis. The ceRNA network identified several molecules (2 long noncoding RNAs, 50 miRNAs, and 49 mRNAs) interacting with* Acin1*, among which miR-674-5p was highly expressed in all sample tissues, and higher than that of other differentially expressed miRNAs, and significantly downregulated in HCC. Multiple online databases such as miRWalk also predicted that miR-674-5p targets *Acin1*. This shows that miR-674-5p may be an important molecule for targeting* Acin1*.

**Conclusion:**

*Acin1* is overexpressed in HCC, and the overexpressed *Acin1* is most likely regulated by miR-674-5p and other ceRNA molecules.

**Main Points**

- Compared with the paracancerous group and healthy control group, the expression of apoptotic chromatin condensation inducer 1 (*Acin1*) was significantly upregulated in hepatocellular carcinoma (HCC) (*P *< .001).
- The top 10 upregulated genes closely related to *Acin1* were *Slc3a2*, *Wiz*, *Srrm2*, *Akt1*, *Hnrnpu*, *Sap18b*, *Pabpn1*, *Ddx39b*, *Eif4a3*, and* Rnps1*. These genes are primarily involved in signaling pathways such as messenger RNA (mRNA) surveillance, RNA transport, spliceosome, Janus kinase/signal transducers and activators of transcription signaling pathway, apoptosis, and ubiquitin-mediated proteolysis.
- The ceRNA network analysis revealed that *Acin1* interacted with several molecules as competing endogenous RNAs (ceRNAs), including 2 long noncoding RNAs, 50 microRNAs (miRNAs), and 49 mRNAs. Among them, miR-674-5p was highly expressed in all sampled tissues, surpassing the expression of other differentially expressed miRNAs, and significantly downregulated in HCC. Various online databases, such as miRWalk, also predicted the targeting of *Acin1* by miR-674-5p. This suggests that miR-674-5p may be an important molecule for targeting *Acin1*.

## Introduction

Hepatocellular carcinoma (HCC) is one of the most common and deadly malignant tumors in the world, ranking third in terms of mortality among all cancers.^1^ It poses a significant threat to human health. At present, with the development of multi-omics research technology, a large number of differentially expressed molecules have been found in HCC tumor tissues and surrounding tissues. They have important significance for cell proliferation, migration, apoptosis, and epithelial–mesenchymal transformation.^2-4^ However, the mechanisms by which these differentially expressed molecules are up- or downregulated are not well understood. In recent years, relevant factors involved in messenger RNA (mRNA) stability, splicing, and translation have been proved to play an important role in the occurrence and development of tumors.^5^ Apoptotic chromatin condensation inducer 1 (Acin1) is an RNA-binding protein, also known as Acinus, which was initially found to induce caspase-3 activation to promote apoptotic chromatin condensation and densification, thereby participating in nuclear fragmentation during apoptosis.^6,7^ It was later found that this protein was also a component of the splicing-dependent multi-protein exon junction complex (EJC) deposited on spliced mRNAs and was involved in splicing-related mRNA metabolism. It plays an important role in posttranscriptional regulation of genes and the expression of specific genes by downregulating nuclear mRNA splicing through the spliceosome, where the spliceosome machinery stops and prevents or reduces mRNA splicing.^8,9^ Recent studies have revealed that *Acin1* plays a role in the physiology and pathology of a variety of diseases and cancers. Lin et al^6^ found by whole transcriptome analysis that the expression of *Acin1* transcript decreased and the splicing patterns changed during the development of brown adipose tissue. This change of *Acin1* transcript differentially regulated the formation of brown adipose tissue in fibroblasts. In 2018, Xue et al^8^ found that the expression level of *Acin1* mRNA in platelets of lung cancer patients was significantly higher than that of healthy control group, and the change in its level had potential clinical value for the diagnosis of lung cancer. In addition, the fusion of the *Acin1*-*Nutm1* gene may also cause cryptic chromosomal rearrangements, which play an important role in infant acute lymphoblastic leukemia.^10^ It is evident that *Acin1 *has an increasingly obvious role in cancer.

However, the involvement of *Acin1* in HCC has not been reported yet, so this study analyzed the expression of *Acin1* mRNA in HCC by whole transcriptome sequencing, bioinformatics analysis, and reverse transcription-quantitative polymerase chain reaction (RT-qPCR), further screened the differentially expressed *Acin1*-related genes in tumor tissues, constructed protein–protein interaction (PPI) and competitive endogenous RNA (ceRNA) regulatory networks, etc., and further analyzed their functions and interactions through online databases to predict the targeting and regulation of *Acin1*. We will further screen the differentially expressed *Acin1*-related genes in tumor tissues, construct PPI and competing endogenous RNA (ceRNA) regulatory networks, analyze their functions and interactions, and further predict the miRNAs targeting *Acin1* through online databases to elucidate the possible molecular mechanisms of *Acin1* gene aberrant expression in HCC. 

## Materials and Methods

### Construction of Hepatocellular Carcinoma Mouse Model, Samples Sequencing, and Data Analysis

One hundred healthy mice aged 6-8 weeks and weighing 25-30 g were used to construct the mouse model of HCC according to the previous method in our laboratory.^11^ The tumor tissues (set up as group “H”) and paracancerous liver tissues (set up as group “C”) were collected separately, as well as liver tissues from control mice injected with saline (set up as group “OO”). Three biological replicates of 100 mg of each sample were taken, and total RNA was extracted using a RNA extraction kit and sent to BGI Genomics Co., Ltd. (hereinafter referred to as “Huada Gene”). The sequencing data were analyzed online using Dr. Tom multi-omics data mining platform (https://biosys.bgi.com/). This study was approved by the Ethical Committee of Youjiang Medical University for Nationalities (Ethics Review No. 20201205026, Date: 2020-12-05).

### Reverse Transcription-Quantitative Polymerase Chain Reaction to Detect the Relative Expression of *Apoptotic Chromatin Condensation Inducer 1*

Total RNA from each sample tissue was reverse transcribed to cDNA and then subjected to real-time fluorescence PCR. The qPCR procedure included pre-denaturation at 95°C for 30 seconds, denaturation at 95°C for 5 seconds, annealing/extension at 60°C for 30 seconds, for a total of 40 cycles. The lysis curve reaction program was set up according to the recommended program for the Roche LightCycler96 RT-qPCR instrument, and relative expression values were calculated using 2^-ΔΔCt^. The expression histograms were plotted using GraphPad Prism version 8.0. The internal reference of RT-qPCR was *Gapdh*. The primer sequences of *Acin1* and *Gapdh* are as follows: the forward primer of *Acin1* was 5”- GACTCTTCCGTAAGACTTAAGG-3:” and the reverse primer was 5”- TTCTCTTCGTTCTTCGC-3”; the forward primer of Gapdh was 5”- GTTGTCTCCTGCGACTCA-3” and the reverse primer was 5”- TGGTCCAGGTTCTTACTC-3”.

### Apoptotic Chromatin Condensation Inducer 1-Related Gene Screening

Differentially expressed *Acin1*-related genes were screened by the Dr. Tom multi-omics data mining platform to construct PPI networks and ceRNA regulatory networks. The screening thresholds for differentially expressed genes were: |log2 (FPKM ratio)| > 1, *Q *< .05 (*Q* is the corrected *P* value and FPKM, fragments per kilobase of transcript per million fragments mapped). The FPKM refers to reads per kilobase of exon model per million mapped reads, that is, the number of reads per 1000 exons per 1 million reads on the pair. The number of reads per kilobase of exon model per million mapped reads.

### Functional Enrichment of Apoptotic Chromatin Condensation Inducer 1-Related Genes and Protein Interaction Network Map Construction

Using Dr. Tom multi-omics data mining platform, we constructed a heat map of the expression of *Acin1* and its related genes in tumor tissues, paracancerous tissues, and healthy mouse liver tissues, analyzed the ceRNA regulatory network and PPI network of *Acin1* and its related genes, and performed Gene Ontology and Kyoto Encyclopedia of Genes and Genomes enrichment analysis on *Acin1* and its related genes. The PPI network maps were further visualized using the String online database (http://string.embl.de/).

### Prediction of microRNAs Targeting the Regulation of Apoptotic chromatin condensation inducer 1

MiRWalk (http://mirwalk.umm.uni-heidelberg.de/), Starbase (https://starbase.sysu.edu.cn/), miRecords (http://mirecords.biolead.org/), and TargetScan (http://www.targetscan.org/) were used to predict miRNAs targeting *Acin1*, and the predicted results were intersected using the jvenn online tool (http://jvenn.toulouse.inra.fr/app/example.html) to screen for possible miRNAs. Prediction conditions: *Acin1* as target gene, selected mouse species, other conditions set by default. The results obtained were further analyzed in combination with the expression of miRNAs in the sequencing data.

### Statistical Analysis

Statistical Package for the Social Sciences Statistics version 23.0 statistical software (IBM Corp., Armonk, NY, USA) was used to analyze the measurement data. The measurement data were expressed as mean ± SD. After the normal distribution and variance homogeneity test, the data meeting the normal distribution and variance homogeneity were tested by 2 independent samples *t*-tests, and the difference was statistically significant with *P *< .05.

## Results

### Expression of Apoptotic Chromatin Condensation Inducer 1 Gene

**Figure 1.**
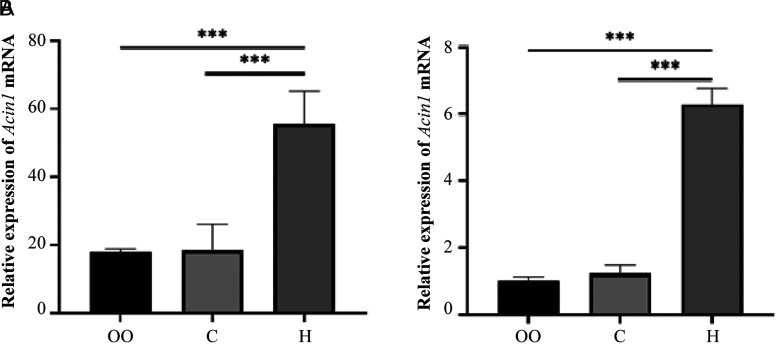
Relative expression of Acin1 mRNA in different tissues. Note: (A) shows the relative expression of Acin1 in the sequencing data; (B) shows the experimental validation of RT-qPCR. group OO: the liver tissue of control mice; group C: the paracancerous tissues of HCC; group. H: the tumor tissue. ***P < .001, **P < .01, * *P *< .05. Acin 1, apoptotic chromatin condensation inducer 1; HCC, hepatocellular carcinoma; mRNA, messenger RNA; RT-qPCR, reverse transcription-quantitative polymerase chain reaction.

The results of sequencing data showed that *Acin1* was expressed in all sample tissues, and *Acin1* mRNA was significantly upregulated in tumor tissues compared with paracancerous tissues and healthy control tissues (*P *< .001). The expression of *Acin1* mRNA was further verified by an RT-qPCR experiment. The results showed that *Acin1* mRNA was also significantly upregulated in tumor tissue (*P *< .001) (Figure 1).

### Interaction Networks and Expression of Apoptotic Chromatin Condensation Inducer 1-related Genes

**Figure 2.**
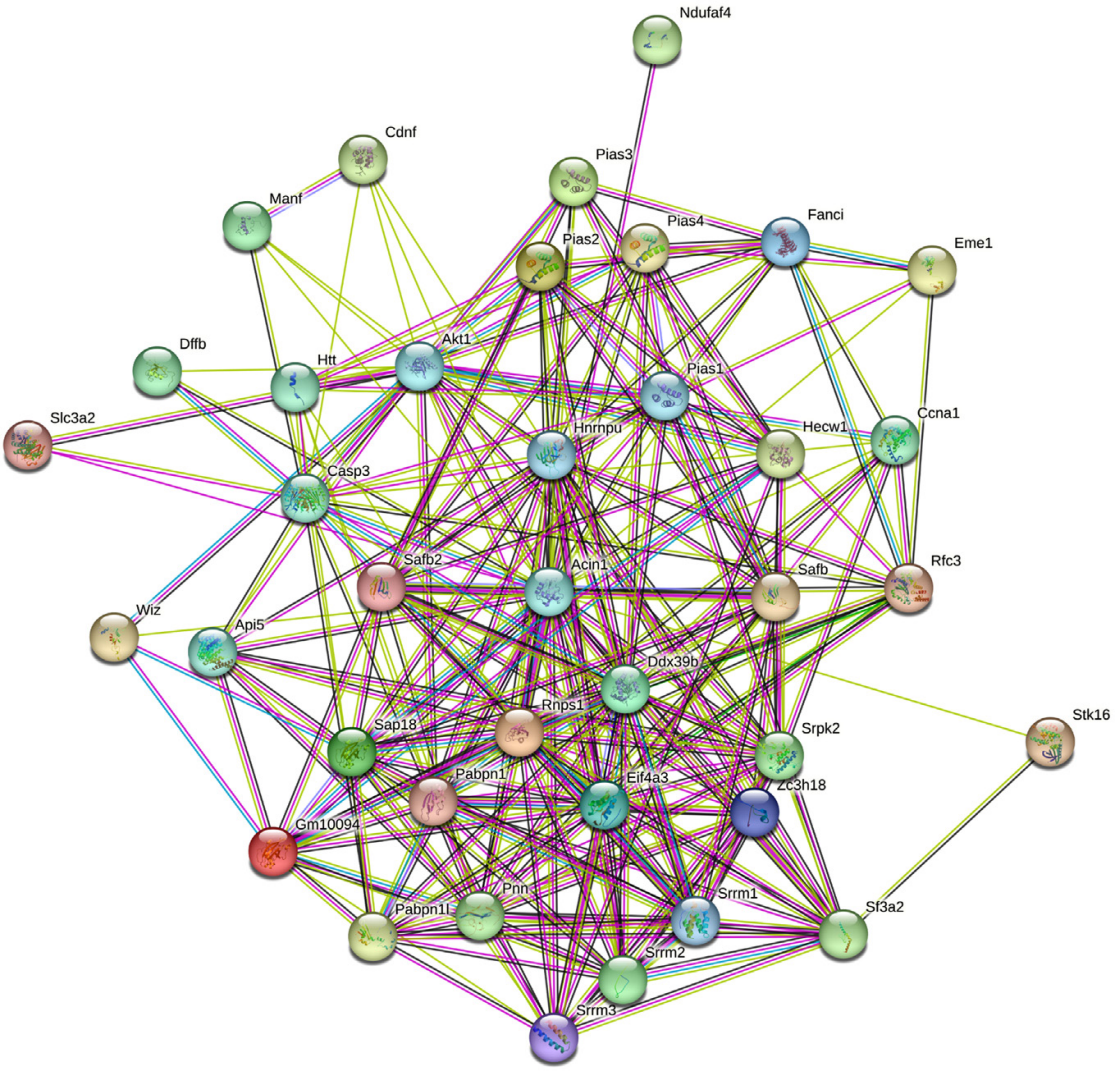
The protein–protein interaction network of apoptotic chromatin condensation inducer 1 and its related genes.

**Figure 3.**
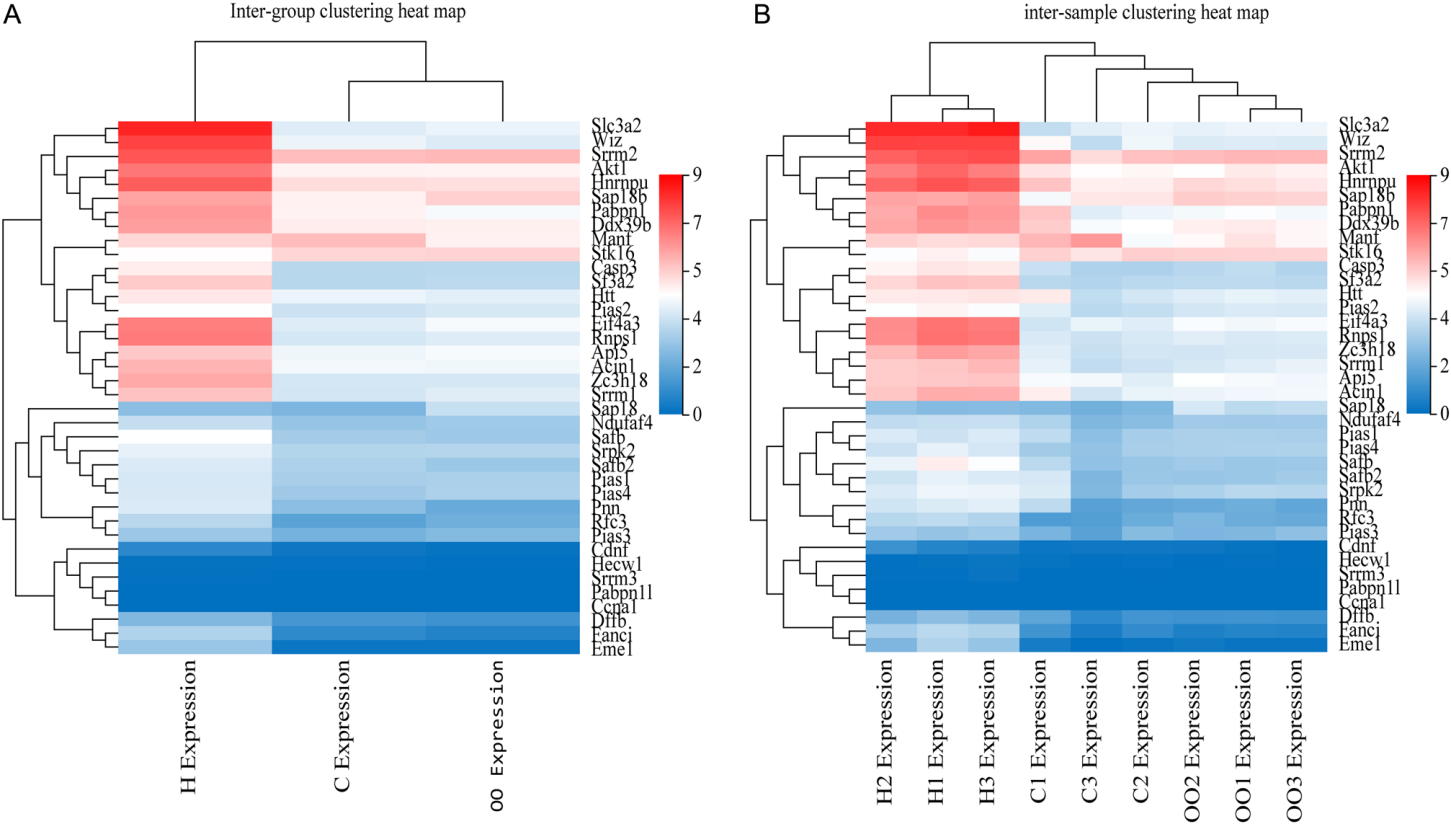
Inter-group clustered heat map (A) and inter-sample clustered heat map (B) of apoptotic chromatin condensation inducer 1 and its related genes. Note: The expression in this figure refers to the fragments per kilobase of transcript per million fragments mapped value.

The results of interaction analysis of *Acin1* and its related genes showed that there were a large number of proteins/genes with PPI relationship with *Acin1*, including 37 closely related proteins/genes (Figure 2). The clustered heat map showed that among the 37 genes, *Slc3a2*, *Wiz*, *Srrm2*, *Akt1*, *Hnrnpu*, *Sap18b*, *Pabpn1*, *Ddx39b*, *Eif4a3*, *Rnps1*, *Api5*, *Zc3h18,* and *Srrm1* were significantly upregulated in tumor tissues compared with those in the paracancerous tissues and healthy control tissues. The intergroup clustered heat map (Figure 3A) and inter-sample clustered heat map (Figure 3B) of the expression of *Acin1* and its related genes were generally consistent (Figure 3), and the top 10 *Acin1*-related genes expressed in HCC are *Slc3a2*, *Wiz*, *Srrm2*, *Akt1*, *Hnrnpu*, *Sap18b*, *Pabpn1*, *Ddx39b*, *Eif4a3,* and *Rnps1*. It can be seen that these 10 genes may play an important role in the regulation of the occurrence and development of HCC by *Acin1*.

### Functions and Pathways Involved in Apoptotic Chromatin Condensation Inducer 1 and its Related Genes

**Figure 4.**
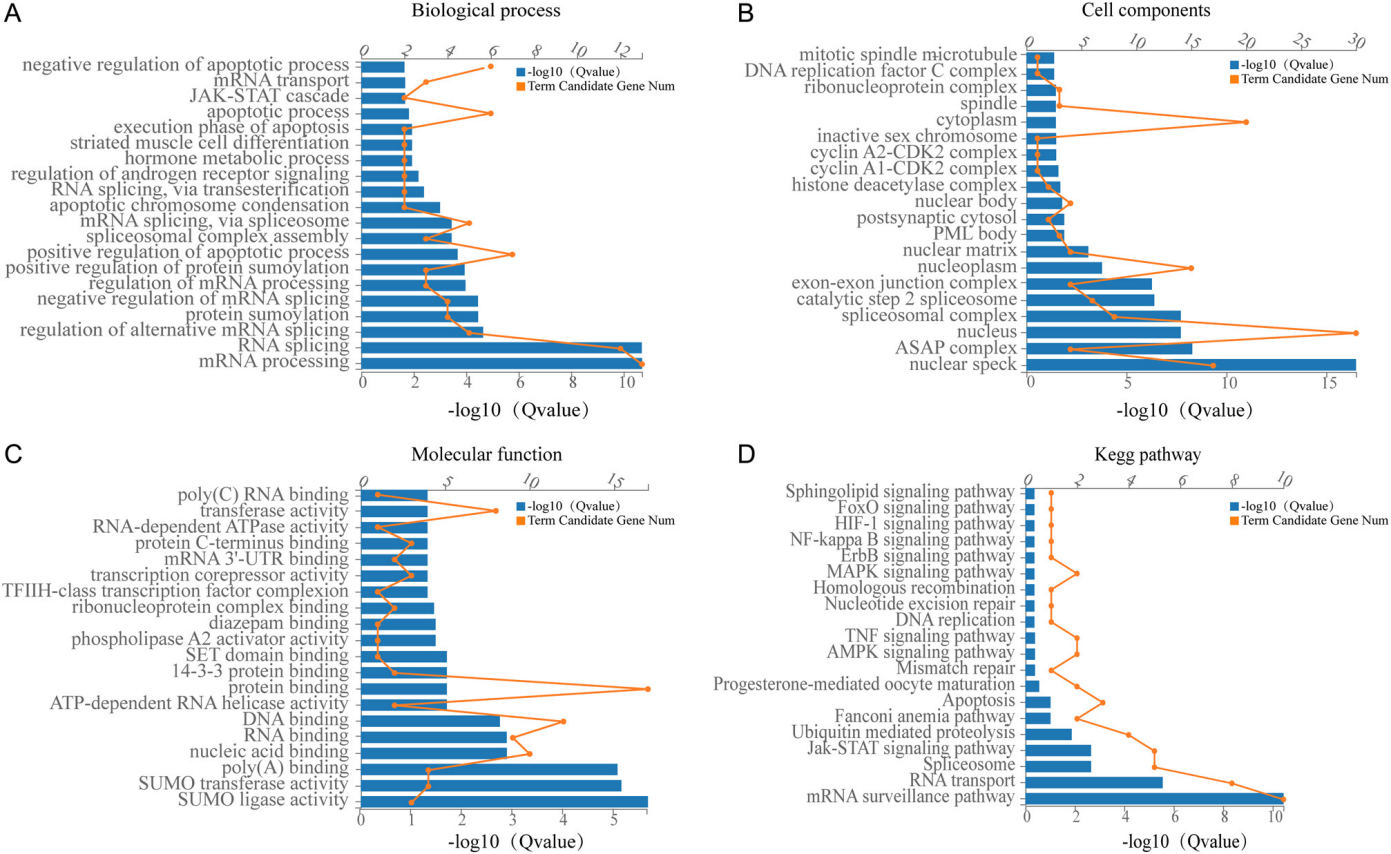
Enrichment analysis of GO and KEGG of *Acin1* and its related genes. Note: A: biological process; B: cellular component; C: molecular function; D: KEGG pathway. Acin 1, apoptotic chromatin condensation inducer 1; GO, gene ontology; KEGG, Kyoto Encyclopedia of Genes and Genomes.

The functional enrichment analysis of *Acin1* and its related genes found that they mainly play a role in the nuclear speck, nucleus, nucleoplasm, spliceosomal complex, EJC, and cytoplasm, etc., participate in biological processes such as mRNA processing, RNA splicing, spliceosomal complex assembly and apoptosis regulation, etc., and play a role in small ubiquitin-related modifier (SUMO) transferase activity, SUMO ligase activity, poly(A) binding, nucleic acid binding, and nucleotide binding. The results of pathway enrichment showed that they were mainly involved in mRNA surveillance, RNA transport, spliceosome, Janus kinase/signal transducers and activators of transcription (JAK-STAT) signaling pathway, apoptosis, and ubiquitin-mediated proteolysis (Figure 4).

### MicroRNAs and Competing Endogenous RNA Network Targeting and Regulating *Apoptotic Chromatin Condensation Inducer 1*

**Figure 5.**
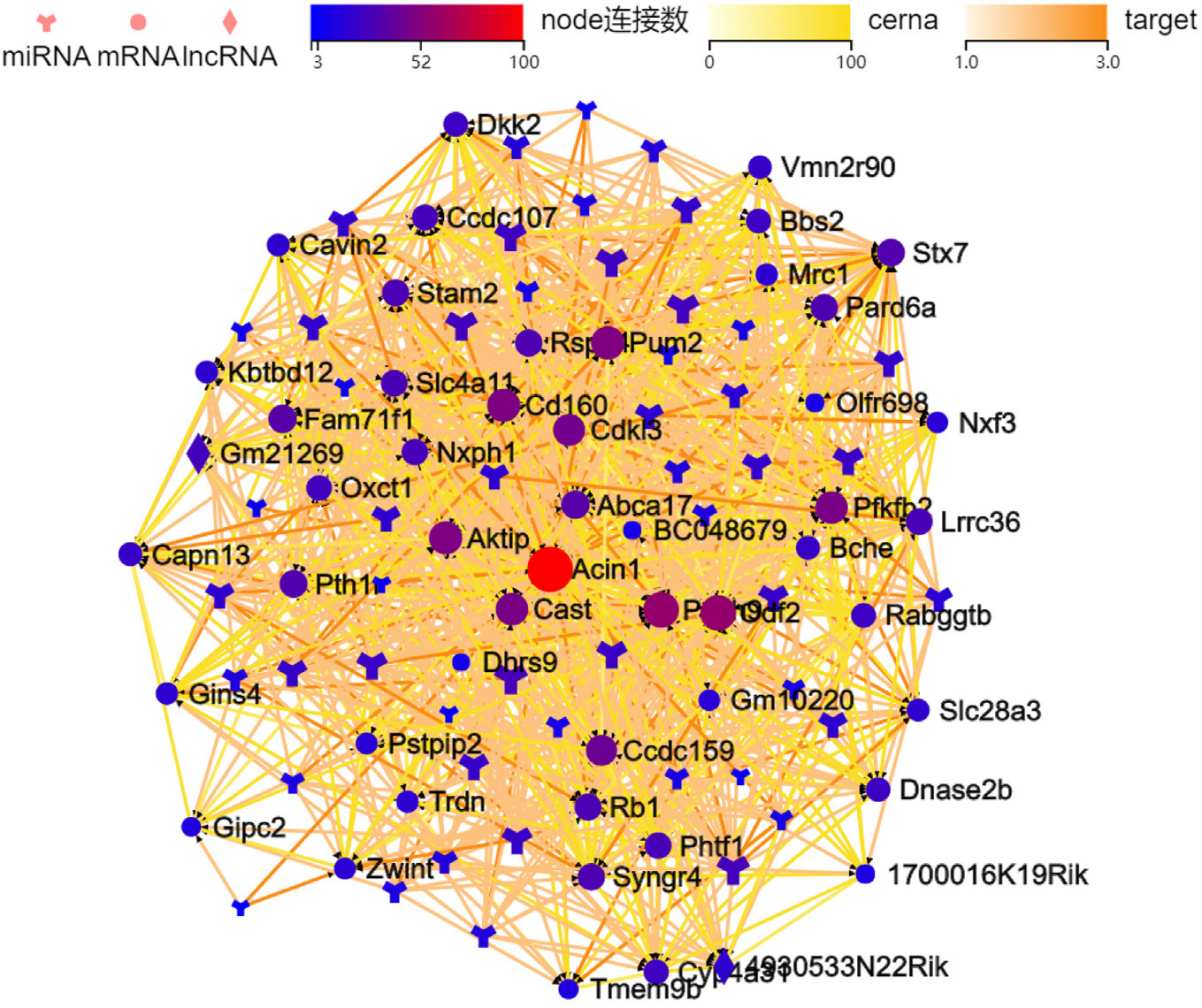
The competing endogenous RNA network of apoptotic chromatin condensation inducer 1 gene and its related genes in hepatocellular carcinoma. lncRNA, long noncoding RNA; mRNA, messenger RNA; miRNA, microRNA.

This study further analyzed the miRNAs that target to regulate *Acin1* and ceRNAs that can competitively combine with miRNA and mRNA to regulate *Acin1*. Through the analysis of the ceRNA regulatory network, we found 101 ceRNAs molecules related to *Acin1*, including 2 long noncoding RNAs (4930533N22Rik and Gm21269), 50 miRNAs, and 49 mRNAs (Table 1). Many molecules have targeted regulatory relationships (Figure 5). Among many miRNAs targeted to regulate *Acin1*, we found 3 miRNAs that were significantly negatively correlated with the expression of *Acin1*, namely miR-6395, miR-674-5p, and miR-7067-5p, of which miR-674-5p was expressed at a higher level in all tissue samples, and its expression was much higher than other differentially expressed miRNAs and was significantly downregulated in tumor tissues relative to paracancerous and healthy control tissues.

**Figure 6.**
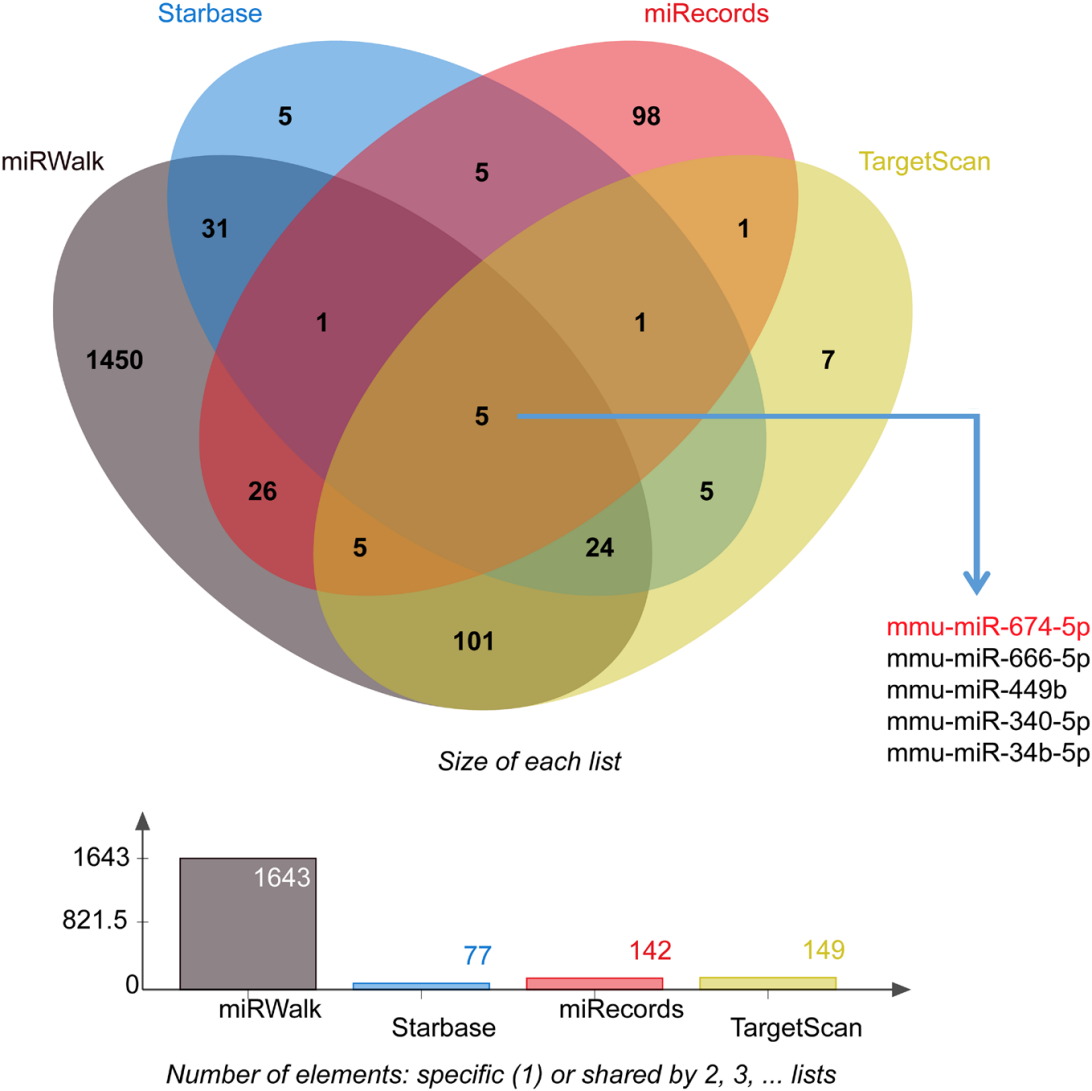
Intersection of microRNA predictions targeting apoptotic chromatin condensation inducer 1 from 4 online databases. Note: The 4 online databases are miRWalk, StarBas, miRecords, and TargetScan online databases.

Further, we used 4 online databases, miRWalk, StarBase, miRecords, and TargetScan, to comprehensively predict the miRNAs targeting *Acin1*, and found that all 4 databases also predicted miR-674-5p targeting *Acin1* (Figure 6). Therefore, we speculate that miR-674-5p may be an important regulator of *Acin1* overexpression, and that multiple ceRNA molecules in the ceRNA regulatory network that are associated with *Acin1* may also play an important role in its overexpression. These molecules may work coordinately to finely regulate *Acin1* expression. 

## Discussion

Genetic and acquired changes in selective splicing of mRNA precursors play an important role in the development of human disease and many cancer-related genes are regulated by alternative splicing.^12,13^ Understanding abnormal splicing or splicing variation will help us understand the causes of malignant transformation. Apoptotic chromatin condensation inducer 1 is a multifunctional nuclear protein, an auxiliary component of the splicing-dependent multiprotein EJC, deposited at the splice junctions of mRNAs, which confers the binding of RNA to the complex and has an important role in apoptosis, selective RNA splicing, and basal autophagy.^14^ It has been shown that the molecule is widely present in a variety of tissues and organs, including glandular vesicles, the retina, blood, lungs, liver,^15-19^ etc. Our results also found that *Acin1* was expressed in liver tissue and was significantly overexpressed in HCC tumor tissue. We know that splice errors that lead to the production of abnormal transcripts rarely occur in normal cells, but research has found that they are almost the inherent characteristics of cancer cells.^20^ Tumor-specific splicing alterations are created by mutations that disrupt splicing-regulatory elements within genes and impair splicing recognition or by altering the RNA-binding preferences of individual splicing factors.^21^ Apoptotic chromatin condensation inducer 1 has the function of participating in splicing-related mRNA metabolism, but it is rarely reported that *Acin1* participates in HCC. In this article, we explore the causes and possible molecular mechanisms of the overexpression of *Acin1* in HCC in order to provide some insights into the pathogenesis of HCC.

Through whole transcriptome sequencing and RT-qPCR, we found that *Acin1* was significantly upregulated in tumor tissues compared with normal control liver tissues and paracancerous tissues (*P *< .001). In order to further understand the cause of its overexpression, we analyzed the Dr. Tom multi-omics data mining platform provided by BGI Genomics Co., Ltd. and identified numerous genes/proteins that interacted with Acin1. Among them, there are 37 genes/proteins closely related to Acin1, and the top 10 genes, *Slc3a2*, *Wiz*, *Srrm2*, *Akt1*, *Hnrnpu*, *Sap18b*, *Pabpn1*, *Ddx39b*, *Eif4a3,* and *Rnps1*, have the highest levels of expression. Further analyzing the function of *Acin1* and its related genes, we found that they are mainly enriched in the cell components such as nuclear speck, nucleus, nucleoplasm, spliceosomal complex, EJC, and cytoplasm and have molecular functions such as SUMO transferase activity, SUMO ligase activity, poly(A) binding, nucleic acid binding, and nucleotide binding. Messenger RNA surveillance, RNA transport, spliceosome, JAK-STAT signaling pathway, apoptosis, and ubiquitin-mediated proteolysis are the main signaling pathways they are involved in. However, the abnormal activation of RNA splicing body and proliferation-related signal pathway, such as JAK-STAT signal pathway, has been repeatedly reported to be closely related to the occurrence and development of tumor.^22-24^ Further analysis of the functional enrichment results revealed that *Acin1*, together with eukaryotic translation initiation factor 4A3 (*Eif4a3*), DEAD box helicase 39b (*Ddx39b*), heterogeneous nuclear ribonucleoprotein U (*HnrnPU*), poly(A) binding protein, nuclear 1 (*Pabpn1*), serine/arginine repetitive matrix 2 (*Srrm2*), and RNA-binding protein with serine rich domain 1 (*Rnps1*), are involved in mRNA processing and RNA splicing together. Together with thymoma viral proto-oncogene 1 (*Akt1*), *Acin1* are also involved in poly(A) RNA binding. In addition, *Acin1,* together with *Eif4a3* and *Rnps1,* also regulates selective mRNA splicing via splice vesicles. It is evident that these *Acin1*-related genes are essential for the functional role of *Acin1*.

MicroRNAs are a class of non-protein-coding regulatory molecules that have been increasingly reported being directly or indirectly involved in the development of many cancers, including liver cancer.^25-27^ In order to explore the cause of the overexpression of *Acin1* in HCC, we further analyzed the miRNAs that target to regulate *Acin1* and ceRNAs that can competitively combine with miRNA and mRNA to regulate *Acin1*. The results showed that many miRNAs can target and regulate *Acin1*. Among them, we found that the expression of miR-674-5p in all sample tissues was higher, and its expression was much higher than other differentially expressed miRNAs and was significantly downregulated in tumor tissues relative to paracancerous and healthy control tissues. This is negatively correlated with the expression of *Acin1*. Further, we also predicted that miR-674-5p is the miRNA that targets and regulates *Acin1* by using several classic miRNA online prediction databases such as miRWalk, StarBas, miRecords, and TargetScan. Therefore, we speculated that miR-674-5p may play a crucial role in the pathogenesis of HCC by targeting *Acin1*. However, further experiments are warranted to confirm the regulatory relationship between miR-674-5p and *Acin1* in HCC development. It has been reported that miR-674-5p/5-LO axis can be involved in the autoimmune response to acute liver injury in mice induced by cutanocin A (cona). This study indicated that the expression of miR-674-5p was significantly downregulated in the liver damaged by cona.^28^ In our study, miR-674-5p is downregulated in HCC, while *Acin1* is upregulated in HCC, which shows that miR-674-5p is likely to regulate the upregulation of *Acin1*. However, the functional role of genes is also often influenced by multiple interacting molecules. In order to further study the possible molecular mechanism of *Acin1* participating in HCC, we also investigated the effect of ceRNA. The ceRNA regulatory network plays an important role in HCC.^29^ In our study, multiple molecules were found to interact with *Acin1* as ceRNAs, including 2 lncRNAs (4930533N22Rik and Gm21269), 50 miRNAs, and 49 mRNAs. These findings suggest that the regulatory mechanism of ceRNA may be significant in the upregulation of *Acin1* and possibly contribute to its fine-tuned regulation.

In summary, this study revealed that *Acin1* is overexpressed in HCC through the utilization of a mouse model of HCC, whole transcriptome sequencing, bioinformatics analysis, and RT-qPCR experiments. It was found that the upregulation of *Acin1* may be regulated by miR-674-5p and multiple ceRNA molecules. They may contribute to the development of HCC by exerting molecular functions such as protein binding, nucleic acid binding, and ubiquitination-like modifications, affecting pathways such as mRNA surveillance, RNA transport, spliceosome, JAK-STAT signaling pathway, apoptosis, and ubiquitin-mediated proteolysis. However, the specific mechanisms through which miR-674-5p and these ceRNA molecules regulate the involvement of *Acin1* in HCC necessitate further investigation in order to obtain a comprehensive understanding of their roles in HCC development.

## Figures and Tables

**Table 1.** The Competing Endogenous RNA Molecules Related to Apoptotic Chromatin Condensation Inducer 1

Type	Number	Gene Symbol
lncRNA	2	4930533N22Rik, Gm21269
miRNA	50	miR-6934-3p, miR-1249-5p, miR-7090-5p, miR-6912-5p, miR-6999-3p, miR-6945-5p, miR-7029-3p, miR-7682-3p, miR-7021-3p, miR-7684-5p, miR-6953-3p, miR-6918-5p, miR-7222-3p, miR-7016-5p, miR-7044-3p, miR-6904-5p, miR-6395, miR-6982-5p, miR-7235-5p, miR-12198-3p, miR-6988-5p, miR-665-5p, miR-6982-3p, miR-6927-5p, miR-6997-5p, miR-7067-5p, miR-1247-3p, novel-miR410-5p, novel-miR114-3p, novel-miR332-3p, miR-674-5p, novel-miR16-3p, novel-miR325-3p, novel-miR5-5p, novel-miR47-5p, novel-miR320-3p, novel-miR308-5p, novel-miR159-3p, novel-miR311-5p, miR-666-5p, miR-7047-5p, miR-8119, miR-6935-5p, miR-6973b-3p, miR-7070-5p, miR-6965-5p, miR-6396, miR-7034-3p, miR-6914-5p, miR-6345
mRNA	49	*Stx7, BC048679, Syngr4, Cyp4a31, Pard6a, Ccdc159, Aktip, Cdkl3, Vmn2r90, Dnase2b, Zwint, Gins4, Rspo1, 1700016K19Rik, Bche, Ccdc107, Slc28a3, Slc4a11, Cd160, Cavin2, Oxct1, Gipc2, Nxph1, Abca17, Olfr698, Kbtbd12, Mrc1, Tmem9b, Trdn, Gm10220, Fam71f1, Rabggtb, Pfkfb2, Pstpip2, Dkk2, Cast, Capn13, Pth1r, Phtf1, Stam2, Rb1, Odf2, Pum2, Dhrs9, Lrrc36, Bbs2, Pcdh9, Nxf3*

lncRNA, long noncoding RNA; miRNA, microRNA; mRNA, messenger RNA.
